# Tertiary lymphoid structures in head and neck squamous cell carcinoma improve prognosis by recruiting CD8 ^+^ T cells

**DOI:** 10.1002/1878-0261.13403

**Published:** 2023-03-08

**Authors:** Mengyao Wang, Rundong Zhai, Mengqi Wang, Weiwen Zhu, Jiayi Zhang, Miao Yu, Wei Zhang, Jinhai Ye, Laikui Liu

**Affiliations:** ^1^ Jiangsu Key Laboratory of Oral Diseases Nanjing Medical University China; ^2^ Department of Basic Science of Stomatology The Affiliated Stomatological Hospital of Nanjing Medical University China; ^3^ Department of Periodontology The Affiliated Stomatological Hospital of Nanjing Medical University China; ^4^ Depatment of Oral and Maxillofacial Surgery The Affiliated Stomatological Hospital of Nanjing Medical University China

**Keywords:** CD8^+^ T cells, head and neck squamous cell carcinoma, lymphotoxin α, tertiary lymphoid structure, tumour microenvironment

## Abstract

Tertiary lymphoid structures (TLSs) are formed in long‐term chronic inflammation, promoting the local recruitment of lymphocytes, antigen presentation and regulation of immune response, correlated with a better prognosis for cancer patients. Although studies have been conducted to explore methods that accelerate the establishment of TLSs, related research in head and neck squamous cell carcinoma (HNSCC) is still lacking. In this study, we analysed data from The Cancer Genome Atlas and performed immunohistochemical staining analyses of 188 patient samples. The results showed that TLSs promoted the infiltration of immune cells. Patients with TLSs with high infiltration of CD8^+^ cells showed the best prognosis. Since lymphotoxin α (LTα) was significantly increased in tissues with TLSs, we overexpressed LTα in SCC7 cells (a mouse‐derived HNSCC cell line) and established tongue‐tumour‐bearing models. The polychromatic observation of tissue sections showed that T‐cell aggregation increased in the LTα cell group, and a grade 1 TLS was formed on the 12th day after inoculating the cells. Moreover, the tumour volume in the LTα group was significantly less than that of the control group, whereas both the number and the proportion of infiltrated CD8^+^ T cells were increased. The peripheral CD8^+^ cells in mice were removed, and no difference was observed in tumour size or TLS formation. Remarkably, we found that TLS led to an increase in the antitumour effect by recruiting CD8^+^ T cells in HNSCC, showing a CD8^+^ T‐cell‐dependent antitumour effect. Moreover, LTα overexpression in the tumour promoted the formation of TLSs.

## Graphical abstract

The authors demonstrate the prognostic value and antitumour function of tertiary lymphoid structures (TLSs) in head and neck squamous cell carcinoma. The crucial role of CD8^+^ T cells was confirmed throughout the development of TLSs and their antitumour process, providing further information to help optimise tumour immunotherapy.

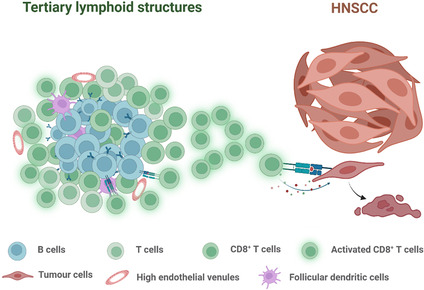

**Abbreviations**
CCK‐8: cell counting kit‐8
CI: confidence interval
DFS: disease‐free survival
FDCs: follicular dendritic cells
H&E: haematoxylin–eosin
HEV: high endothelial veins
HNSCC: head and neck squamous cell carcinoma
HR: hazard ratio
IFN: interferon
IHC: immunohistochemistry
IL‐2: interleukin‐2
LTα: lymphotoxin α
LTβ: lymphotoxin β
LTβR: lymphotoxin‐beta receptor
mIHC: multiple immunohistochemistry
OS: overall survival
PNAd: peripheral lymph node vascular addressing
ROC: receiver‐operating characteristic
RT: room temperature
RT‐qPCR: RNA extraction and quantitative reverse transcription PCR
SLO: secondary lymph nodes
ssGSEA: single‐sample gene set enrichment analysis
TEX: exhausted T cells
Tfh: follicular helper T
TIL: tumour infiltrating lymphocytes
TLS: tertiary lymphoid structure
TME: tumour microenvironment
TNF: tumour necrosis factor
TNFSF14: the 14th member of the tumour necrosis factor superfamily
Tregs: regulatory T cells

## 1 Introduction

Head and neck squamous cell carcinoma (HNSCC) is an aggressive malignancy with poor prognosis. In 2021, about 878 000 people suffered from and 444 000 died of HNSCC [1]. At present, surgical treatment is the preferred therapy. For patients with metastasis and advanced stage of the disease, radiotherapy and chemotherapy are the main treatment methods; however, the prognosis is not satisfactory [2]. One of the reasons is that the cancer cells reduce inherent immunogenicity and the expression of certain chemokines, promoting proliferation and immune regulation of regulatory T cells (Tregs). This leads to the inhibition of CD8^+^ cytotoxic T cells [3]. The immune escape mechanisms increase treatment complexity, especially in the advanced disease stage. Nowadays, with the rapid development of immunotherapies, such as anti‐PD‐1 therapy and anti‐CTLA‐4 [4], several strategies are being explored to resolve the immunosuppressive state. Increasing tumour‐infiltrating lymphocytes (TILs) infiltration and regulating tumour immunosuppression are of great significance [5]. However, as the structure of tumour microenvironment in HNSCC is still unclear, the problem of achieving the immune‐active state is a concern for several scholars.

Recently, the immunomodulatory effect of tumour‐associated tertiary lymphoid structures (TLSs) was largely reported. TLSs are characterised by B‐cell and T‐cell aggregation areas with high endothelial veins (HEVs) around, similar to secondary lymphoid organs (SLOs) [6]. TLSs develop in regions with chronic immune activation, such as cancer, autoimmune disease and chronic inflammation. The antigen is presented by B cells and follicular dendritic cells (FDCs) in mature TLS, and peripheral lymph node vascular addressing (PNAd) is expressed through the surrounding HEV to recruit primitive and memory T cells, thus upregulating the local immune response [7, 8]. A large number of studies have reported that TLSs are important prognostic factors of tumours, including breast cancer, melanoma, liver cancer and lung cancer [9, 10, 11, 12]. The formation of TLSs is the result of the accumulation of TILs, and TLS can further promote this process and enhance the antitumour response. A few studies have proposed an immune subtype that predicts disease‐free survival (DFS), characterised by the formation of TLSs and the infiltration of CD8^+^ and CD4^+^ T cells without the expression of inhibitory receptors [13]. In hepatocellular carcinoma, the density of peritumoural TLSs is an independent prognostic factor. A high density of peritumoural TLSs is usually accompanied by the upregulation of IL‐6, which can inhibit the differentiation of Tregs and promote an active antitumour response [14]. Although the prognostic value of TLSs in HNSCC has been reported [15, 16], the relationship between TLSs and TILs is still unclear.

The development of TLS provides a new direction to optimise the tumour microenvironment and improve treatment effects. Targeting LIGHT, the 14th member of the tumour necrosis factor superfamily (TNFSF14), to blood vessels can promote the formation of HEV and establish TLS in mouse pancreatic cancer by interacting with the lymphotoxin‐beta receptor (LTβR) of endothelial cells, which has a better antitumour effect combined with anti‐PD‐1 and anti‐CTLA‐4 therapies [17]. There is still a lack of an animal model of tumour with TLS, especially to study HNSCC.

Lymphotoxin α (LTα), also known as TNFβ, is a cytokine that belongs to the family of lymphotoxins, which exist either as soluble or as membrane‐bound molecules [18]. Both forms can accelerate the infiltration of immunocytes; however, the soluble form was reported to have the tumour‐killing effect [19]. LTα plays a key role in lymph node development. In a study, the maturation of SLOs in LTα gene knockout mice was inhibited [20]. Antibody‐LTα fusion proteins were also reported to accelerate the infiltration of T cells, promoting the development of TLS and antitumour immune response in melanoma [18]. Whether it can help establish TLS in HNSCC needs further research.

Here, we explored the existence, characteristics and impact of TLSs in HNSCC, and established TLS by overexpressing LTα in a tumour‐bearing model to identify new ideas for the optimisation of the treatment.

## 2 Materials and methods

### 2.1 Public data processing and analysis

The haematoxylin–eosin (H&E) staining of formalin‐fixed paraffin embedded slices of The Cancer Genome Atlas (TCGA)‐HNSCC cases were obtained from (https://cancer.digitalslidearchive.org/), and the assessment of TLS in tumour environment was performed by two pathologists after excluding the cases with low‐quality image acquisition or no corresponding TCGA RNA‐seq data. Single‐sample gene set enrichment analysis (ssGSEA) scores of two different gene signatures that were reported for TLS evaluation were compared by receiver‐operating characteristic (ROC) analysis [21, 22, 23]. The one showed greater predictive ability was used for the follow‐up analysis.

The gene expression profiles of 546 HNSCC patients were obtained from the TCGA database. After excluding 44 normal cases, we predicted the TLS enrichment score and categorised the HNSCC cases into low TLS score group, medium TLS score group and high TLS score group through unsupervised clustering. ssGSEA were used to evaluate the relationship between TLS and 29 immune‐related gene sets. Furthermore, we calculated the immune score, matrix score, immune mechanism score and tumour purity [24], and obtained the abundance of 22 different tumour‐infiltrating immune components using CIBERSORT [25].

### 2.2 Patients and samples

In this study, paraffin‐embedded tissues of 188 patients were obtained from the Stomatological College of the Nanjing Medical University between 2010 and 2015. Both clinical and pathological diagnosis of these patients was HNSCC, and the cancer sites included tongue, the floor of mouth, cheek, gingiva, palate and oropharynx. Complete clinical and prognostic information and well‐preserved pathological tissue were also obtained.

The study was approved by the Ethics Committee of Nanjing Medical University (2019; #848). According to the institutional guidelines, all participants or their relatives signed informed consent documentation, and the study protocol was in accordance with the Helsinki declaration.

### 2.3 Haematoxylin–eosin staining

All the tissues were cut into serial sections. The sections were dewaxed and rehydrated by decreasing the concentration of alcohol to water. For H&E staining, the sections were washed with PBS thrice and stained with haematoxylin dye solution for 5 min. Then, the slices were stained with eosin dye solution for 1 min.

### 2.4 Immunohistochemical staining and multiple immunohistochemical staining

For immunohistochemical (IHC) staining, the dewaxed and rehydrated sections were washed with PBS thrice. Microwave antigen repair was performed with a citric acid solution. After that, the sections were washed with PBS and then cultured in 3% hydrogen peroxide at room temperature (RT). Furthermore, the sections were washed and the non‐specific antibodies were blocked with goat serum. The sections were incubated with primary antibodies at 4 °C overnight. After incubation, the sections were washed and incubated with secondary antibodies at RT for 1 h. After washing, the samples were stained with the chromagen 3,3′‐diaminobenzidine and haematoxylin.

For the IHC evaluation, we first observed the samples using low‐power (100×) magnification and selected the five areas with a high density of positively stained cells to photograph them using the high‐power (400×) magnification. The positively stained cells were counted in each image, and the average value of the five measurements was used for further analyses. The procedure was carried out by two experienced pathologists. The observers reached an agreement by re‐evaluation and discussion if their evaluation was different.

The multiple immunohistochemical (mIHC) staining was performed using the PANO 4‐plex kit (Panovue, Beijing, China). Briefly, after dewaxing, hydration, antigen repair, non‐specific antibody block and incubating primary and secondary antibodies, PPD520, PPD540, PPD570, PPD650 and tyramide signal amplification reagents were used for amplifying stained signals.

### 2.5 TLS evaluation

First, we observed the distribution of TLS using H&E staining. The structures where lymphocytes gathered without clear boundaries were defined as immature TLS. Mature TLS was characterised by a clear germinal centre. The cases in which only mature TLS or mature and immature TLS were observed were recorded as mature TLS^+^, while those with only immature TLS were recorded as immature TLS^+^. The others were recorded as TLS^−^.

To further identify TLS, IHC staining of HEV markers PNAd, B‐cell marker CD20, and T‐cell marker CD3 was performed using the serial sections. The aggregation of CD20^+^ B cells and CD3^+^ T cells with HEV around was defined as TLS. According to the aggregation pattern of CD20^+^ B cells and the formation of B cell follicular structure, TLS was categorised into three grades: Grade 1 TLS: T cells concentrated in the centre and B cells aggregated or scattered around the T cell area was recorded as 1 point; Grade 2: B cells and T cells gathered into clusters and the boundary between them was unclear was recorded as 2 points; while Grade 3 TLS, which was also mature TLS, showed that B cells infiltrated into the centre and formed follicular structures with T cells surrounding it was recorded as 3 points. All grades of TLS in the whole section were recorded separately, and the total score in each case was calculated after correlating it with the results of H&E staining. TLS evaluation was performed by two experienced pathologists. If the observers made different judgements on the TLS classification of the same case, they reached an agreement through reassessment and discussion.

### 2.6 RNA extraction and quantitative reverse transcription PCR (RT‐qPCR)

The total RNA from the tissue was extracted using Trizol reagent (Life Technologies, Carlsbad, CA, USA) and reverse‐transcribed into cDNA using PrimeScript RT Master Mix (Takara, Shiga, Japan). Furthermore, cDNA was used to perform qRT‐PCR using SYBR Green Mastermix Kit (Vazyme, Nanjing, China). All the primers for qRT‐PCR were listed in Table S1.

### 2.7 Cell lines and lentivirus transduction

SCC7, a mouse‐derived HNSCC cell line, was donated by Q. Wang (Yijishan Hospital of Wannan Medical College, Wuhu, China) and cultured in RPMI‐1640 medium (GIBCO, Grand Island, NY, USA) containing 10% FBS and 1% penicillin–streptomycin. All cells were verified by STR genotyping and were routinely tested for mycoplasma at regular intervals throughout the whole course of the study. The cells were infected with lentiviral particles (Genechem, Shanghai, China) carrying the mouse Ltα gene to establish the SCC7 Ltα overexpression cell line.

### 2.8 Cell counting kit‐8 assay

To examine cell proliferation after transfection, SCC7 cells were cultured in a 96‐well plate at 6 × 10^3^ cells/well. The medium in each well was replaced by a fresh medium containing 10% cell counting kit‐8 (CCK‐8) solution (ApexBio, Houston, TX, USA) for the following 4 days. After a 2‐h incubation, the absorbance was measured at 450 nm using a microplate reader (Molecular Devices, Sunnyvale, CA, USA).

### 2.9 Wound‐healing assays

SCC7 cells were seeded in the six‐well plate until 90% confluence was attained. A scratch was made on the bottom of each well. After washing with PBS, cells were imaged at 0 and 24 h.

### 2.10 Animal models

C57BL6 mice (5–8 weeks old, male) were purchased from Experimental Animal Center of Nanjing Medical University (Nanjing, China) and raised under specific pathogen‐free conditions in the Animal Core Facility of Nanjing Medical University. Animals from each cage were randomly allocated to the control or treated groups. The SCC7 cells were centrifuged, resuspended in RPMI‐1640 medium and injected into the tongues of the mice (5 × 10^6^ per mouse). On the seventh day, five mice of each group were sacrificed, and the tongue volume was measured. On the 12th day, all mice were sacrificed, and the tongues were taken out for specimen fixation and observation. For CD8^+^ T‐cell depletion, mice were treated with inVivoMAb anti‐mouse CD8α (Bio X Cell, Lebanon, NH, USA). The antibody was injected intraperitoneally the day before SCC7 injection, and then injected every 3 days.

All animal procedures were carried out with the approval from the animal ethics committee of the Nanjing Medical University (IACUC‐2101038).

### 2.11 Flow cytometry

On the seventh day after the injection of SCC7 cells, the mice were sacrificed, and the tumour tissues were extracted to make a single‐cell suspension. The mouse TILs were isolated from the tumours using the tumour lymphocyte infiltration kit (Solarbio, Beijing, China), stained with APC‐conjugated anti‐CD3, PE‐conjugated anti‐CD8 and PerCP‐conjugated anti‐CD4 (Biolegend, San Diego, CA, USA), and detected using flow cytometry.

For the apoptosis assay, SCC7 cells were harvested and detected using Annexin V‐PE/7‐AAD staining assay and flow cytometry.

### 2.12 Statistical analysis

All statistical analyses were performed using ibm spss statistics (version 22.0) (IBM Corporation, Armonk, NY, USA) and r‐4.2.1 for windows. A *k*‐fold cross‐validation strategy (*k* = 5) was used for the internal validation to validate the performance of the signature. 95% Confidence intervals (CIs) of AUC were calculated after 1000 bootstrap resampling. Student's *t*‐test and Mann–Whitney *U*‐test were used to evaluate the difference in variables between the two groups, and a chi‐squared test was used to evaluate the difference in variables across the three groups. Pearson's correlation analysis was performed to evaluate the correlation between TLS abundance and infiltration of different immune cell populations in all tumour types. For survival analysis, overall survival (OS) was measured from the date of first diagnosis to death. Patients alive at the last follow‐up date were censored. DFS was measured from the date of diagnosis to the local recurrence, distant metastases or death. The earliest date of any one of them happened would be set as the ending point. Patients alive with disease‐free status were censored at the last date of follow up. Kaplan–Meier (KM) analysis, cox univariate and cox multivariate model were used for survival analysis. Log rank test was used to compare the survival curves. Statistical significance was set at *P*‐value < 0.05.

## 3 Results

### 3.1 High‐density TLS gene signature correlated with tumour immunity

The TLS of 40 HNSCC cases from TCGA were evaluated and TLS were found in 18 cases. According to ROC curve analysis, the gene signature containing 12 chemokines [22, 23] had better prediction effect, which would be used for following analysis, whose AUC value was 0.699 (95% CI 0.507–0.831, *P* = 0.019), and the mean AUC of the fivefold cross‐validation was 0.732 (Fig. S1A).

**Fig. 1:**
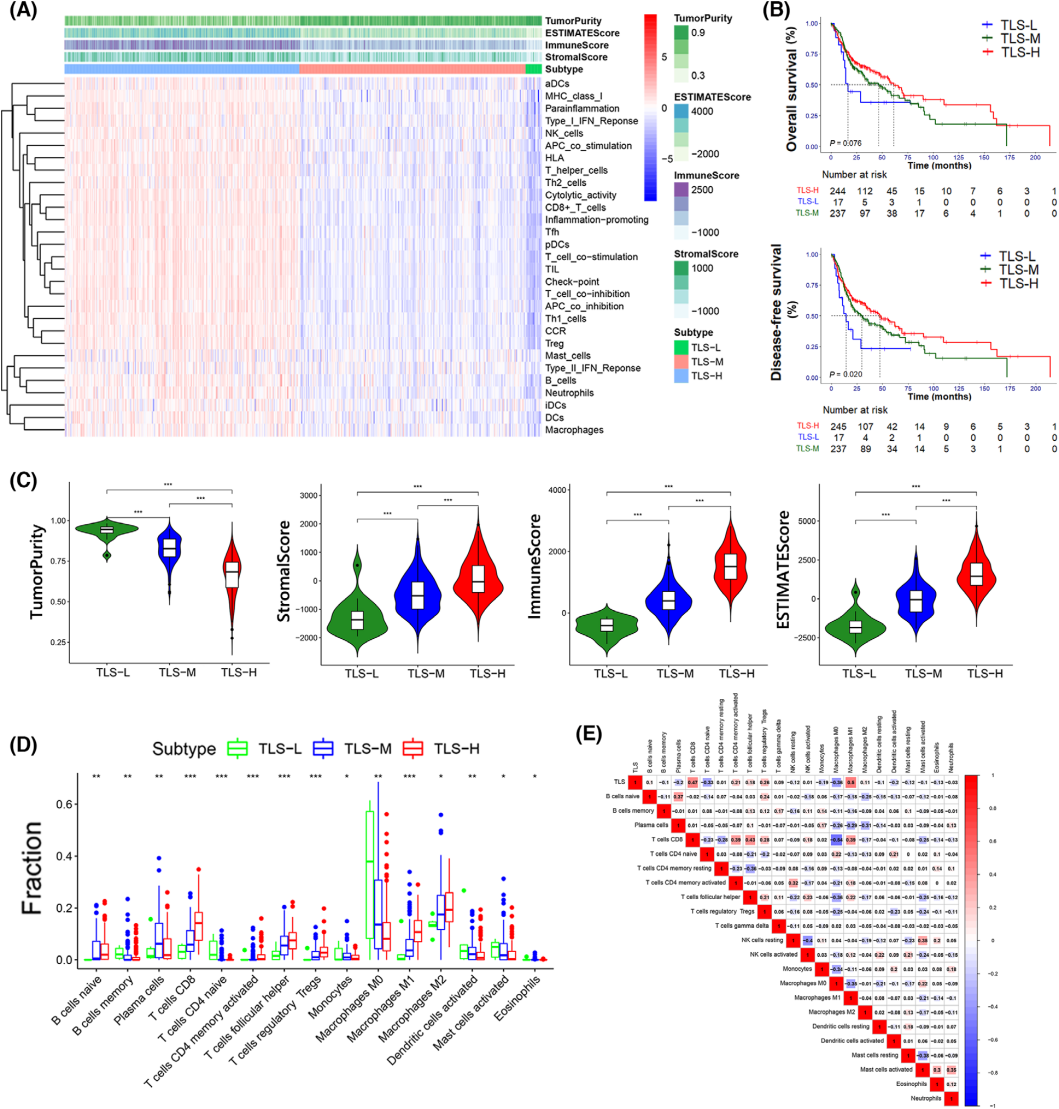
Correlation between TLSs, immune status and prognosis. TLS‐L indicates low TLS score group, TLS‐M indicates medium TLS score group, and TLS‐H indicates high TLS score group. (A) The ssGSEA score in 29 immune genes set of each sample were showed in the heatmap with the estimate results shown on the top. (B) Kaplan–Meier survival analysis of the TLS‐score related subtypes. The *P*‐value and HR of the pairwise comparisons were showed in Table S2. (C) The tumour purity, stromal score, immune score and estimate score in different subtypes. The horizontal lines indicate median values, boxes indicate 25% and 75% quartiles, and error bars represent minimum/maximum values. The asterisks indicate the *P*‐value: ***< 0.001 (Mann–Whitney *U*‐test). (D) The relative fractions of 15 immune cells in different TLS related subtypes. The horizontal lines indicating median values, boxes indicating 25% and 75% quartiles, and error bars represented minimum/maximum values. The asterisks indicate the *P*‐value: *< 0.05; **< 0.01; ***< 0.001 (Mann–Whitney *U*‐test). (E) The correlation analysis of TLS score and 22 immune cells.

A total of 546 patients were screened from the TCGA‐HNSC project, and 502 cases of cancer RNA‐seq data were obtained. Through unsupervised clustering using 12 TLS‐related chemokines, the cases were divided into three groups: low TLS score group (17 cases), medium TLS score group (238 cases) and high TLS score group (247 cases) (Fig. 1A). Significant correlation between TLS and tumour size was showed by cross‐analysis (Table 1). The survival analysis showed that the OS and DFS of the high, medium and low score groups decreased gradually, and the *P*‐value was close to the critical value (Fig. 1B, Table S2). Multivariate Cox regression analysis showed that TLS score was a protective factor of OS [hazard ratio (HR) = 0.142 (95% CI 0.024–0.858), *P* = 0.033] (Fig. S1B).

**Table 1:** Cross‐analysis of clinical information and TLS enrichment group (TCGA). Results are based on nonempty rows and columns in each innermost subtable.

	TLS cluster						*P*‐value
	TLS‐H		TLS‐M		TLS‐L		
	Count	Per cent	Count	Per cent	Count	Per cent	
Gender							
Male	172	69.90	181	76.40	14	82.40	0.192
Female	74	30.10	56	23.60	3	17.60	
Age							
< 60	101	41.10	111	46.80	9	52.90	0.336
≥ 60	145	58.90	126	53.20	8	47.10	
Location							
Oral cavity	42	17.10	28	11.80	2	11.80	0.394^a^
Oral floor	26	10.60	31	13.10	3	17.60	
Oral tongue	80	32.50	64	27.00	4	23.50	
Oropharynx	73	29.70	90	38.00	5	29.40	
Others	25	10.20	24	10.10	3	17.60	
Stage							
I–II	65	27.30	44	19.00	5	29.40	0.09
III–IV	173	72.70	187	81.00	12	70.60	
T							
T1–T2	100	42.20	70	30.30	6	35.30	0.028*
T3–T4	137	57.80	161	69.70	11	64.70	
N							
N0–N1	147	63.10	160	70.20	12	70.60	0.834^a^ ^,^ ^b^
N2–N3	86	36.90	68	29.80	5	29.40	
M							
M0	230	98.70	223	99.10	17	100.00	0.256
M1	3	1.30	2	0.90	0	0.00	
Grade							
G1–G2	169	71.30	182	79.50	11	64.70	0.077
G3–G4	68	28.70	47	20.50	6	35.30	

^a^ More than 20% of cells in this subtable have expected cell counts < 5. Chi‐square results may be invalid.

^b^ The minimum expected cell count in this subtable is < 1. Chi‐square results may be invalid.

* The chi‐square statistic is significant at the 0.05 level.

To explore the relationship between TLS and tumour immunity, 29 immune‐related gene sets were analysed in HNSCC cases with ssGSEA and the heat map was generated. The results showed that with the increase in TLS score, the enrichment of immune‐related genes increased significantly (Fig. 1A). The immune score, stromal score, estimate score and tumour purity score were calculated, and significant differences were found across the three groups. The high expression group had a higher immune score, stromal score, and estimate score and a lower tumour purity (Fig. 1C).

To explore the correlation between TLS and infiltrated immune cells, we obtained the predicted values of 24 immune cells in the cases through CIBERCORT and found that naïve B, CD8^+^ T, CD4^+^ activated memory T, follicular helper T cells, Tregs, and M1 macrophages significantly increased when TLS enrichment was higher, while memory B cells, naive CD4^+^ T cells, M0 macrophages, and activated dendritic cells decreased in the high TLS score group (Fig. 1D). The correlation analysis was performed between the scores of the immune cells and TLS gene signature scores. It showed that M1 macrophages and CD8^+^ T cells were positively correlated with TLS enrichment score while naïve CD4^+^ T cells and M0 macrophages were negatively correlated (Fig. 1E).

### 3.2 The formation and presence of TLS correlated with better prognosis

**Fig. 2:**
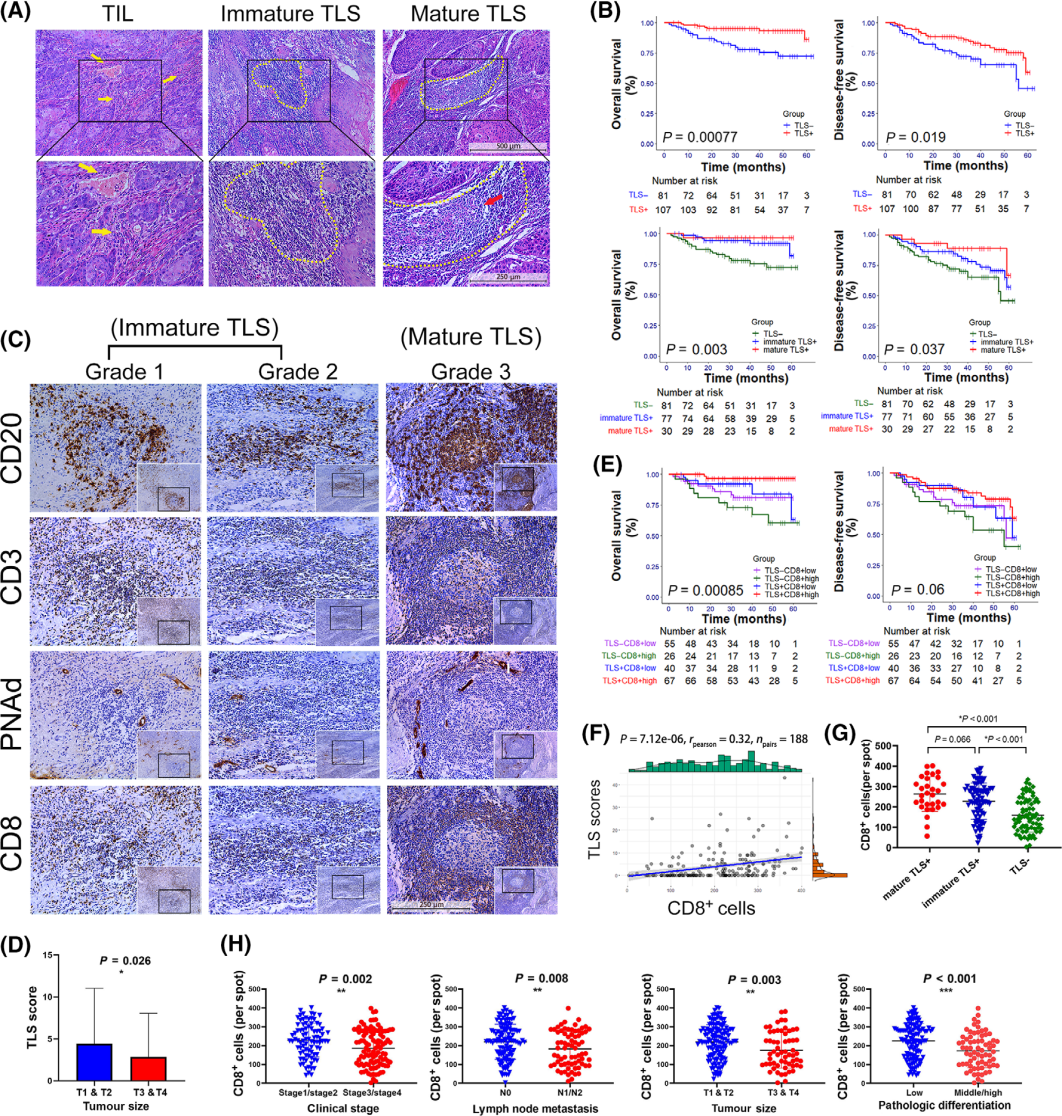
Histological properties and prognostic value of TLSs in HNSCC. (A) Representative H&E staining pictures of TILs, immature TLSs and mature TLSs taken under 100× (upper, scale bar: 500 μm) and 200× (lower, scale bar: 250 μm). The yellow arrow marks indicate TILs, the yellow border marks indicate TLSs, and the red arrow mark indicates the germinal centre of mature TLS. (B) The OS and DFS curves of patients. Patients were divided into two groups according to TLS existence (upper), or three groups according to mature TLS existence and immature TLS existence (lower). The HR for OS (TLS^+^ vs. TLS^−^) was 0.248 (95% CI 0.103–0.598) and the HR for DFS (TLS^+^ vs. TLS^−^) was 0.516 (95% CI 0.294–0.906). The *P*‐value and HR of the pairwise comparisons between the three subgroups were showed in Table S3. (C) Representative pictures of the three grades of TLS taken under 200× (scale bar: 250 μm). The classification was based on the IHC staining of CD20, CD3 and PNAd. The IHC staining of CD8 was performed to show the correlation between CD8^+^ T cells and TLS. (D) TLS scores in different clinical groups. Patients were divided by tumour size. The TLS scores were calculated based on the number of three grades of TLS. The error bars represent the SD of the mean. The asterisks indicate the *P*‐value: *< 0.05 (Mann–Whitney *U*‐test). (E) The OS and DFS curves of patients. Patients were divided into four groups according to TLS existence and CD8^+^ cells. The *P*‐value and HR of the pairwise comparisons between the subgroups were showed in Table S4. (F) The correlation analysis between TLS scores and CD8^+^ cells [*R* = 0.32 (95% CI 0.19–0.44), *P* < 0.001]. (G) CD8^+^ cells in mature TLS^+^ group, immature TLS^+^ group and TLS^−^ group. The error bars represent the SD of the mean. The asterisks indicate the *P*‐value: *< 0.05 (Student's *t*‐test). (H) CD8^+^ cells in different clinical groups. Patients were divided by clinical stage, lymph node involvement, tumour size, pathologic differentiation. The error bars represent the SD of the mean. The asterisks indicate the *P*‐value: **< 0.01; ***< 0.001 (Student's *t*‐test).

The H&E staining of the 188 clinical samples was performed to distinguish TILs, immature TLS and mature TLS (Fig. 2A). In immature TLS, TIL was aggregated while the germinal centre was not formed. B‐cell‐follicular‐germinal centre was formed in mature TLS, with TIL clustered more closely. The aggregation of TIL in mature TLS is more closely, with a germinal centre formed. The boundary between the germinal centre and surrounding lymphocytes was clear (Fig. 2A). Cross‐analysis between TLS and clinical information showed no significant correlation with gender, age, invasive pattern or location (Table 2).

**Table 2:** Cross‐analysis of clinical information and TLS.

	TLS				*P*‐value
	Negative		Positive		
	Count	Per cent	Count	Per cent	
Gender					
Female	36	44.40	41	38.30	
Male	45	55.60	66	61.70	0.398
Age					
< 62	38	46.90	55	51.40	
≥ 62	43	53.10	52	48.60	0.542
Invasive pattern					
Invasive	31	38.30	43	40.20	
Non‐invasive	50	61.70	64	59.80	0.790
Location					
Buccal	25	30.90	28	26.20	
Gingival	16	19.80	13	12.10	
Tongue	23	28.40	39	36.40	
Oral floor	5	6.20	6	5.60	
Others	12	14.80	21	19.60	0.459
Pathologic differentiation					
Low	48	60.00	69	64.50	
Middle and high	32	40.00	38	35.50	0.531
Tumour size					
< 5 cm	48	59.30	80	74.80	
≥ 5 cm	33	40.70	27	25.20	0.024*
Lymph node metastasis					
Negative	49	60.50	67	62.60	
Positive	32	39.50	40	37.40	0.767
Clinical stage					
Stage 1 & 2	33	40.70	53	49.50	
Stage 3 & 4	48	59.30	54	50.50	0.231
PNAd					
Low	53	65.40	42	39.30	
High	28	34.60	65	60.70	< 0.001**
CD20					
Low	58	71.60	36	33.60	
High	23	28.40	71	66.40	< 0.001**
CD3					
Low	49	60.50	40	37.40	
High	32	39.50	67	62.60	0.002**
CD8					
Low	55	67.90	40	37.40	
High	26	32.10	67	62.60	< 0.001**

The *P*‐values were obtained using the chi‐squared test. The asterisks indicate the *P*‐values: *< 0.05; **< 0.01.

Among 188 patients, mature TLS was observed in 30 cases and immature TLS in 106 cases. Single TLS development was hardly to be observed, especially when mature TLS had developed. Immature TLSs usually meanwhile developed when mature TLS existed. Mature TLS appeared alone in only one case. Survival analysis showed that the prognosis of patients with TLS was better than others and the differences in OS and DFS were significant (*P*‐value is < 0.001 and 0.019, respectively) (Fig. 2B). Patients with only immature TLS had better prognosis than those without TLS. The OS and DFS of patients with mature TLS were better than those with immature TLS while the difference was not significant (Fig. 2B, Table S3). Multivariate Cox regression analysis showed that TLS score was an independent protective factor of OS [HR = 0.874 (95% CI 0.765–0.997), *P* = 0.045] (Fig. S2A).

### 3.3 Development of mature TLS went through three grades

**Fig. 3:**
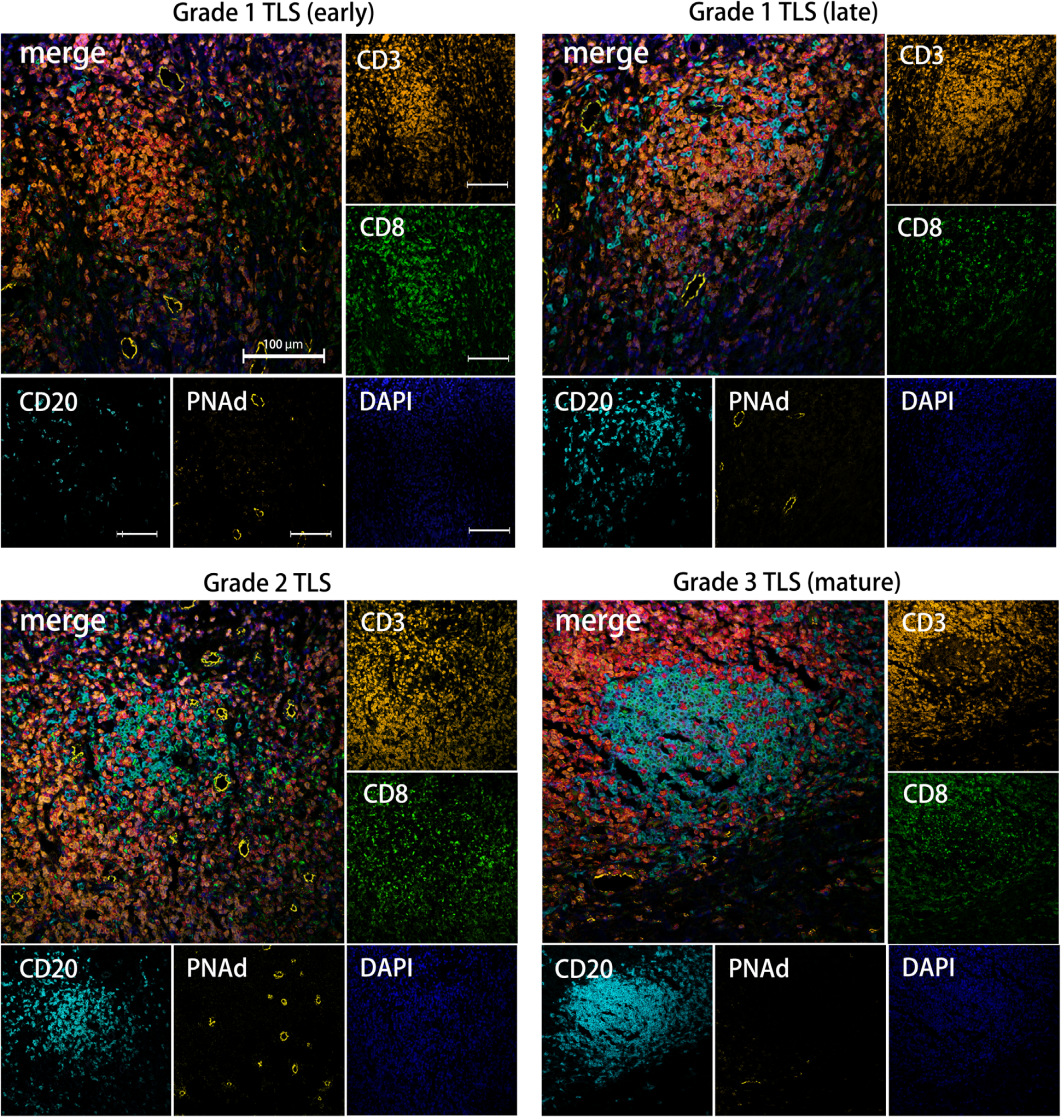
Multiple immunohistochemical staining pictures of TLSs. Representative mIHC pictures of Grade 1 TLS (early state) (upper left), Grade 1 TLS (late state) (upper right), Grade 2 TLS (lower left) and Grade 3 TLS (mature TLS) (lower right) taken under 200× (scale bar: 100 μm).

According to the definition of TLS, the identification of CD20^+^ B cells, CD3^+^ T cells and PNAd^+^ HEV were required for the identification; therefore, the IHC staining of CD20, CD3 and PNAd was performed using the serial sections. The entire sections were observed based on results from H&E staining. It showed that TLSs had different degrees of T‐cell and B‐cell aggregation with PNAd^+^ vessels surrounding them (Fig. 2C). Immature TLS were further classified into two types based on the aggregation characteristics of B cells and T cells. Three grades of TLS were identified according to B cells and T cells clusters using IHC and multi‐IHC, and the results indicated the gradual process of TLS maturity. T cells gathered early to form a T‐cell centre and surrounding B cells began to gather (grade1 TLS). An increasing number of B cells further clustered to the centre (mixed with T cells in grade 2 TLS) until they formed follicle structures (grade 3 TLS), which were similar to that of the SLOs (Figs 2C and 3). With multi‐IHC, we observed early and late states of Grade 1 TLS, which clearly showed the infiltration trade of B cells after T cells clustered (Fig. 3).

Based on the total TLS score of each patient, TLS score was associated with tumour size, but TLS had no relationship with lymphatic metastasis, clinical stages, or pathology stages (Fig. 2D, Fig. S2B).

### 3.4 The correlation of CD8^+^ T cells with a good prognosis was correlated with the TLS score

CD8^+^ T cells were then evaluated using IHC (Fig. 2C). Contrary to the TLS scores, we found that the number of CD8^+^ cells increased significantly in groups with lymph node metastasis, higher clinical grades and pathological grades and bigger tumour sizes (Fig. 2H), but were not associated with invasive pattern and the prognosis (Fig. S2C,D). The correlation analysis revealed that the number of infiltrated CD8^+^ T cells increased in TLS^+^ sections, especially in the m‐TLS^+^ sections, and were significantly associated with the TLS score (Fig. 2F,G). The cases were categorised into four groups, namely TLS^+^ CD8^+^ high, TLS^+^ CD8^+^ low, TLS^−^ CD8^+^ high and TLS^−^ CD8^+^ low. Moreover, the prognostic analysis was performed, and it was found that the TLS^+^ CD8^+^ high group had the best prognosis, while the TLS^−^ CD8^+^ high group had the worst prognosis (Fig. 2E, Table S4).

Matching the same position and performing multi‐IHC, we observed that CD3^+^CD8^+^ T cells had already clustered in the early state of immature TLS, and increased in mature TLS. CD3^+^ CD8^+^ T cells were heavily recruited around the centre of B cells after the formation of the follicle centre (Fig. 3).

### 3.5 The overexpression of Ltα promoted the formation of TLS

We obtained the expression levels of LTα from the TCGA database and found that they were significantly different across the three TLS subtypes (Fig. S3B). The correlation analysis also revealed a good correlation between LTα and TLS gene signatures [*R* = 0.62 (95% CI 0.57–0.67), *P* < 0.001] (Fig. S3A). RNA was extracted from the 21 HNSCC tissue samples, and it was found that the LTα expression significantly increased in the tissues with TLS (Fig. S3C). After the sections were stained, it was found that in TLS^+^ sections, LTα expression in the tumour cells was also significantly stronger (Fig. S3D).

**Fig. 4:**
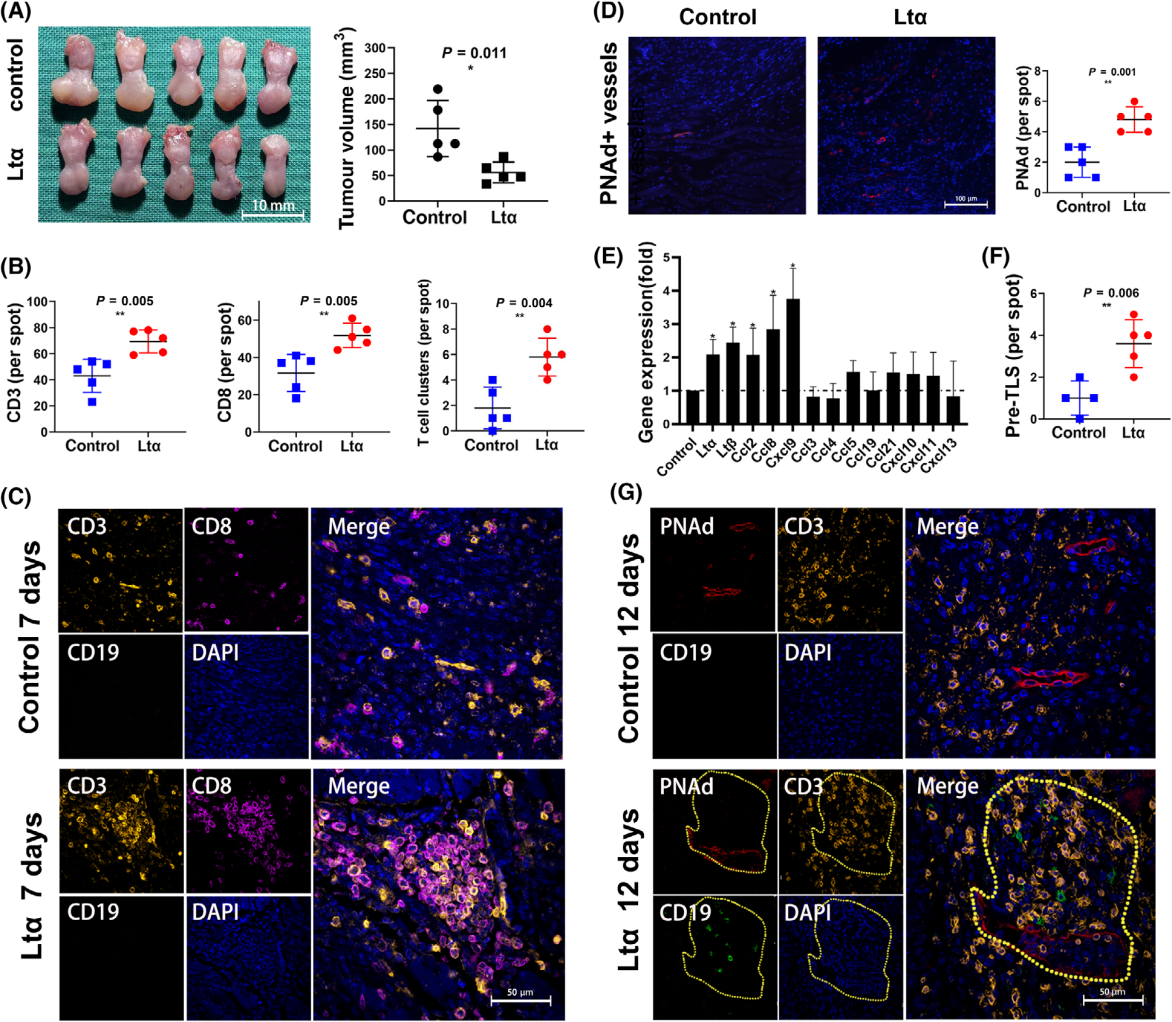
Tongue tumour‐bearing models developed by the injection of SCC7 cells whose Ltα was over expressed. (A) General views of the tongue of the mouse model. The tumour volume was compared in control group (*n* = 5) and Ltα group (*n* = 5) (scale bar: 10 mm). The error bars represent the SD of the mean. The asterisks indicate the *P*‐value: *< 0.05 (Student's *t*‐test). (B) CD3^+^, CD8^+^ cells and T‐cell clusters in control group mice (*n* = 5) and Ltα group mice (*n* = 5) on the seventh day. The error bars represent the SD of the mean. The asterisks indicate the *P*‐value: **< 0.01 (Student's *t*‐test). (C) Representative pictures of multiple immunochemical staining of CD3, CD8 and CD19 in control group mice (*n* = 5) and Ltα group mice (*n* = 5) on the seventh day (scale bar: 50 μm). (D) PNAd staining results in different groups (scale bar: 100 μm). The error bars represent the SD of the mean. The asterisks indicate the *P*‐value: **< 0.01 (Student's *t*‐test). (E) Expression of TLS related mRNA (Ltα, Ltβ, Ccl2, Ccl8, Cxcl9, Ccl3, Ccl4, Ccl5, Ccl19, Ccl21, Cxcl10, Cxcl11, Cxcl13) in tumours from Ltα group (*n* = 5) on the seventh day. The mRNA expression was presented relative to those from control group (*n* = 5) (far left). The error bars represent the SD of the mean. The asterisks indicate the *P*‐value: *< 0.05 (Student's *t*‐test). (F) Pre‐TLS (the Grade 1 TLS‐like‐structure) development in different groups. The asterisks indicate the *P*‐value: **< 0.01 (Student's *t*‐test). (G) Representative pictures of multiple immunochemical staining of CD3, CD8 and CD19 in control group mice (*n* = 5) and Ltα group mice (*n* = 5) on the 12th day (scale bar: 50 μm). The yellow border marks indicate Pre‐TLS.

**Fig. 5:**
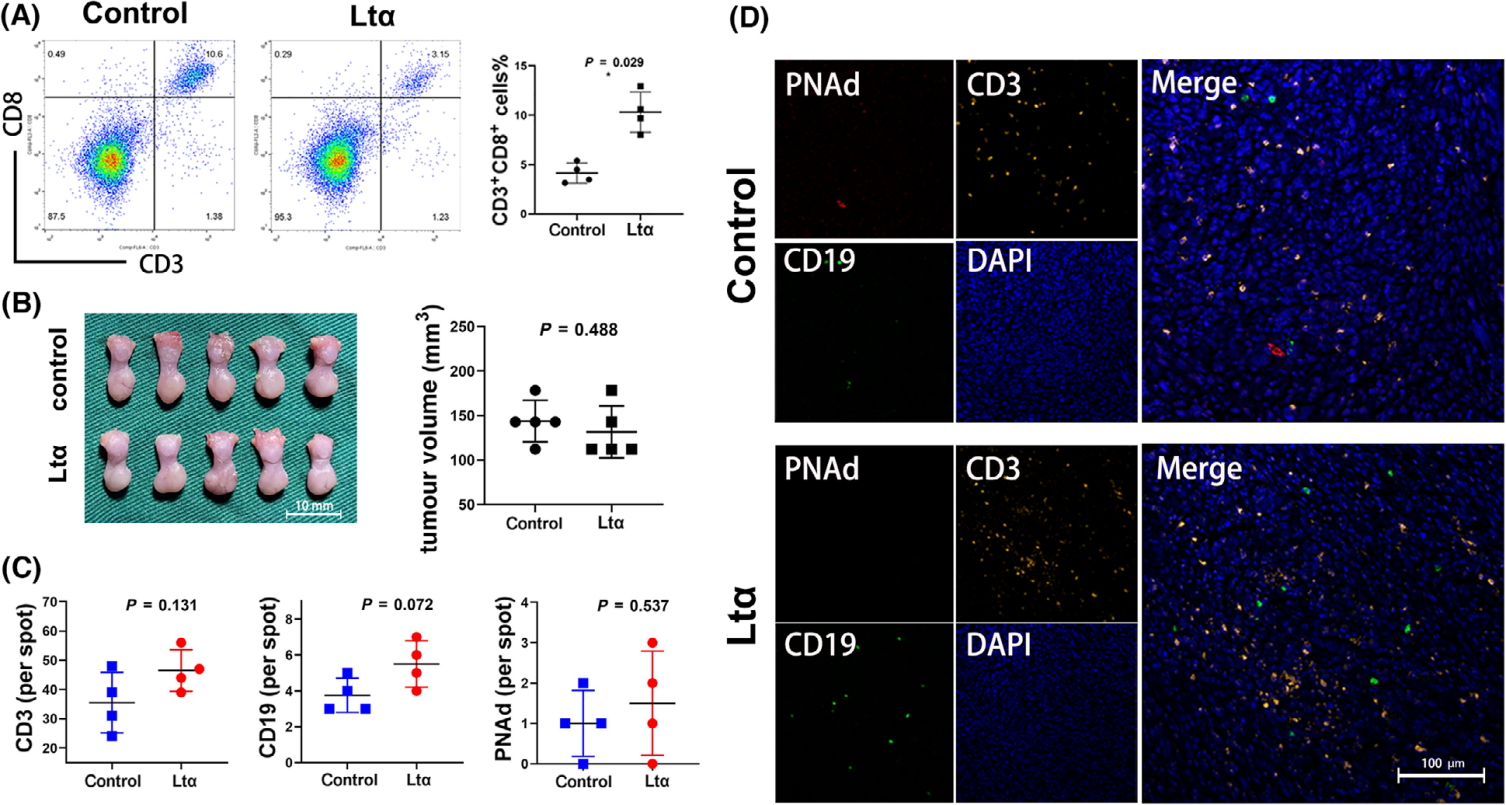
The antitumour effect and pre‐TLS development of Ltα group were blocked by CD8^+^ cells elimination. (A) Frequency of CD3^+^ CD8^+^ T cells among tumour infiltrated lymphocytes from control group mice (*n* = 4) and Ltα group mice (*n* = 4). The error bars represent the SD of the mean. The asterisks indicate the *P*‐value: *< 0.05 (Student's *t*‐test). (B) General views of the tongue of the mouse model after CD8^+^ cells elimination (scale bar: 10 mm) (left). The tumour volume was compared in control group (*n* = 5) and Ltα group (*n* = 5) (right). The error bars represent the SD of the mean. The *P*‐value was obtained using the Student's *t*‐test. (C) CD3^+^, CD19^+^ cells and PNAd^+^ vessels in control group (*n* = 4) and Ltα group (*n* = 4) on the 12th day after CD8^+^ cells elimination. The error bars represent the SD of the mean. The *P*‐values was obtained using the Student's *t*‐test. (D) Representative pictures of multiple immunochemical staining of PNAd, CD3 and CD19 in control group (*n* = 4) and Ltα group (*n* = 4) on the 12th day after CD8^+^ cells elimination (scale bar: 100 μm).

Stable trans‐strains of Ltα‐overexpressing‐lentiviral transfected SCC7 cell lines were screened for further analysis (Fig. S4A,B). The results of the CCK8 proliferation assay showed no significant differences in the proliferative capacity of Lt‐SCC7 and control‐SCC7 (Fig. S4C), as well as in the results of migration ability, cell cycle distribution and cell apoptosis detection (Fig. S4D–F). Therefore, Ltα‐SCC7 and control‐SCC7 were injected into the tongue of the C57BL6 mice, which were sacrificed on days 7 and 12. The formation of oral squamous epithelial carcinoma was confirmed using IHC of PanCK in tissue sections (Fig. S5A). Moreover, we performed IHC of CD19, CD3, and PNAd and observed a significant increase in CD3^+^ T cell infiltration and PNAd^+^ vessel formation in the Ltα‐SCC7 group, while CD19^+^ B cells hardly infiltrated in either of the groups on the seventh day after injection (Fig. 4C,D). The T cells in the Ltα‐SCC7 group already showed a tendency for local aggregation, while those in the control group were mostly scattered around the tumour (Fig. 4B,C). Compared with the control group, the expression of Ltα and lymphotoxin β (LTβ) significantly increased at the Ltα‐SCC7 tumour site on Day 7 (Fig. 4E). Meanwhile, most TLS‐related genes were upregulated to varying levels, especially CCL2, CCL8 and CXCL9 (*P*‐value < 0.05) (Fig. 4E). On the 12th day, the number of CD3^+^ T cells, CD8^+^ T cells, CD19^+^ B cells and PNAd^+^ HEV all increased significantly on the 12th day (Fig. S5C). The Grade 1 TLS‐like‐structure appeared in the Ltα‐SCC7 group, which we defined as pre‐TLS. Pre‐TLS has PNAd^+^ vessels formation around the T‐cell aggregation region, accompanied by the recruitment of a small number of CD19^+^ B cells (Fig. 5G, Fig. S5B).

### 3.6 The formation and antitumour effect of TLS were correlated with CD8^+^ T cells

The tumour size was measured on the seventh day after cell injection, and it was found that compared with the control group, the tumour volume in the Ltα‐SCC7 group was smaller, and the results were statistically significant (Fig. 4A). Furthermore, TILs were extracted from the animal model for flow cytometry, which showed that the proportion of CD3^+^ CD8^+^ T cells in the total lymphocytes increased significantly in the Ltα‐SCC7 group (Fig. 5A). Simultaneously, IHC staining revealed that the CD8^+^ T cells had already clustered at the centre of the pre‐TLS while CD19^+^ B cell started infiltrating, the same as TLS in the clinical samples (Fig. S5B).

To demonstrate the antitumour effect of CD8^+^ T cells and their relationship with TLS, mice were injected with tumour cells after the peripheral CD8^+^ cells were eliminated. By observing the size of the mouse tongue on Day 7, no significant difference in tumour size was found between the two groups (Fig. 5B).

On the 12th day, multi‐IHC results showed that more PNAd^+^ HEV developed, while CD3^+^ cells hardly clustered in the Ltα‐SCC7 and control groups. No significant difference in the T‐cell and B‐cell infiltration number was found between the two groups, and the aggregation of lymphocytes was rarely observed (Fig. 5C,D).

## 4 Discussion

Through the prediction by the public database and the evaluation of clinical samples, the presence of TLS was confirmed in HNSCC. Meanwhile, its role in promoting the infiltration of lymphocytes and the recruitment of CD8 T cells in the tumour immune microenvironment was predicted and verified. Subsequently, to investigate the formation of TLS, we established a mouse tongue tumour model and accelerated the formation of TLS by overexpressing LTα in tumour cells. The local overexpression of LTα accelerated the formation of TLS and showed antitumour effects, both of which were dependent on CD8^+^ T cells.

Head and neck squamous cell carcinoma is one of the most common primary malignancies with a high rate of metastasis and recurrence [26]. Despite improvement in surgical treatment strategies for HNSCC, the prognosis of patients with advanced cancer has not improved. For better efficacy of postoperative chemoradiotherapy or immunotherapy, it is crucial to improve the tumour immune microenvironment [2, 27]. HNSCC is generally considered immunogenic cancer [28]. However, the degree of immune infiltration varied greatly in different patients as shown using both the public data analysis and IHC staining, which may be related to HPV positivity. Compared with HPV^+^ HNSCC, HPV^−^ HNSCC showed more immune infiltration and a higher level of CD8^+^ T‐cell activation, which is thought to be more suitable for immunotherapy [29, 30]. Analysis of public RNA‐seq data revealed that a higher immune score correlated with lower tumour purity and better prognosis. Meanwhile, the TLS gene signature showed superior overall immune status and close correlation with the killer cells, such as CD8^+^ T cells. These results were also confirmed using IHC staining of the pathological sections, suggesting that TLS may improve tumour microenvironment (TME), increase the antitumour effect, and thus, promote prognosis. Accelerating TLS formation could help optimise the treatment for patients with advanced disease.

In recent years, TLS has received much attention in the field of tumour research. Similar to the other tumours reported, mature TLS, which was observed in HNSCC, has similar composition and morphology to secondary lymph nodes [31, 32, 33, 34], including B‐cell germinal centres, surrounding T cells and blood vessels that express PNAd. After entering the TLS, the raw B cells differentiated and matured through their interaction with the follicular helper T (Tfh) cells of follicular DC cells, and eventually formed a follicular‐like B‐cell centre. In addition to being able to act as antigen‐presenting cells, B cells can secrete interferon (IFN)‐γ and IL‐12, to promote cytotoxic CD8^+^ T‐cell response [6]. Meanwhile, the HEV surrounding the TLS expressed the peripheral node addressin [35], recruiting a large number of initial T cells from the peripheral blood, which can be locally activated and differentiated into CD8 T cells in the TLS. Thus, a positive circulation of immune response was achieved.

Several studies classified tumour‐associated TLS into immature and mature TLS [15, 36]. In our study, immature TLS was further categorised into two grades: Grade 1 TLS, which had T‐cell centres; and grade 2 TLS, which had B cell–T cells mixed centre. Grade 3 TLS were mature TLS with B‐cell general centre. Therefore, the process of TLS formation can be considered to be initiated by T‐cell infiltration and the formation of B‐cell germinal centre can be considered as a marker of maturation. Patients with mature TLS showed a better prognosis than immature TLS while the result was not significant that may be caused by the insufficient sample size. Those without TLS showed the worst prognosis, indicating that TLS plays a continuous role in antitumour response during and after the formation, and its antitumour effect is more obvious after maturation. This speculation was confirmed by CD8 staining. CD8 was scattered at the centre of the Grade 1 and Grade 2 TLS, and when the mature TLS was formed, CD8 accumulated in the periphery. This may have resulted due to the improvement in antigen presentation by the mature TLS and the massive recruitment and activation of initial T cells. TLS^+^ patients with more infiltration of CD8^+^ T cells had the best DFS and OS, indicating that TLS was closely related to the killing effect of CD8^+^ T cells. It was also shown that TLS^−^ patients with more CD8^+^ T cells had a poorer prognosis than those with fewer CD8^+^ T cells. Exhausted T cells (TEX) may explain this result. TEX is a subtype of CD8^+^ T cells, initially identified in chronic persistent infections, and also frequently seen in tumour immunity [37]. It loses effector function in a progressive and graded manner, mainly characterised by the production defects in interleukin‐2 (IL‐2), tumour necrosis factor (TNF), and IFN‐γ, resulting in the failure of cytotoxicity. An increase in TEX is often accompanied by a reduction in effector T cells [38, 39]. We predicted that the immunological function of patients with TLS^−^ tumours was depressed and found that IL‐2, TNF and INF‐γ decreased with a decrease in the expression of TLS‐related genes using data analysis, suggesting an increase of TEX. On the contrary, after the formation of the TLS, the activated B cells, Tfh cells, and FDCs in TLS can all present antigens and secrete a variety of cytokines, regulating the balance of T cells [6]. The correlation between TLS and TEX is not clear yet, and the relevant mechanisms need to be further studied.

Lymphotoxin α forms a heterotrimer (LTα2β or LTαβ2) with the LTβ. This heterotrimer can function as a membrane‐binding molecule for signalling through the LTβR pathway, which plays a key role in the formation of lymph nodes [20, 40, 41]. LTα can also form a homotrimer (LTα3) that triggers the transduction of cytotoxic signals through two major receptors, the TNF receptor I (TNFR I; p55 TNFR) and TNFR II (p75 TNFR) [42]. The LTα3 homotrimer can also promote T‐cell homing and induce the production of IgA by B cells [19]. In our research, the direct killing effect of LTα on tumour cells was not significant, and the apoptosis in SCC7 cells overexpressing LTα was not significantly different from that in the control groups in our study. According to the preclinical data, LTα can be synergistic and cytotoxic to epithelial cancer cells when used in combination with chemotherapeutic drugs, especially cisplatin. However, upon systemic use of the LTα derivative rhLTα‐Da, the patient showed significant systemic response due to the proinflammatory effect, presenting with fever, chills, hypertension, etc. [43]. Therefore, we focussed on tumour cells, attempting to explore the feasibility of achieving therapeutic effects by local overexpression of LTα. Through IHC staining, we recognised that the LTα expression of the tumour cells also significantly increased in cases with dense lymphatic infiltration in addition to the stromal cells. The LTα expression showed a gradual increase from the tumour centre to the stroma. CCK8 experiments, invasion experiments and cell cycle detection of SCC7 cells overexpressing LTα showed that it had no obvious effect on the proliferation, invasion, and cell cycle of the tumour cells themselves, respectively. This suggested that our animal experimental results mainly relied on the *in vivo* immune environment.

In the current study, we successfully established TLS *in vivo* by overexpressing LTα in SCC7 cells and confirmed that this method has a potential therapeutic effect. After CD8^+^ T‐cell depletion, the antitumour effect decreased, suggesting that the efficacy of LTα overexpression was dependent on CD8 T cells. Furthermore, CD8 T cells not only infiltrated after TLS development but also acted as an essential parameter during TLS formation. Peripheral B‐cell recruitment and HEV formation were not affected by the deletion of CD8^+^ T cells, but the gathering of local CD45 cells significantly decreased. A decrease in the expression of LTα may explain this result, for CD8^+^ T cells are one of the lymphocytes who express lymphotoxin [44, 45]. Moreover, during early infiltration, some of the CD8^+^ T cells can transform from effector T cells to memory T cells, which secrete chemokines for further recruitment [46]. It has been reported that CD8 T cells in the TLS germinal centre were independent prognostic factors that may promote the cell‐mediated antitumour immune response, but the specific underlying mechanisms need to be explored [47].

The model was established in the tongue of the mice and the growth of tumour may have become a barrier during feeding, leading to emaciation if kept for a long period. Therefore, long‐term tumour‐bearing models cannot be established, which makes it unable to verify the efficacy of local injection of LTα overexpression virus at this stage. Therapeutics to enhance tumour immunity still need more intensive study and validation.

## 5 Conclusions

In this research, we verified that TLS related to the good prognosis of HNSCC, which may result by the increase of CD8^+^ T cells recruitment. We found that local overexpression of Ltα benefited the formation of TLS, the recruitment of CD8^+^ T cells as well as antitumour functions, which provided a new clue for the optimisation scheme of tumour immunotherapy.

## Conflict of interest

The authors declare no conflict of interest.

## Author contributions

Mengyao Wang was involved in conceptualisation, data curation, formal analysis, methodology, software, visualisation, writing—original draft and revision. RZ was involved in conceptualisation, data curation, methodology, software, visualisation, writing—original draft and revision. Mengqi Wang was involved in data curation, methodology, software, supervision, visualisation and revision. WZ was involved in data curation, methodology, software and revision. JZ was involved in data curation, methodology and validation. MY was involved in methodology, software and funding acquisition. WZ was involved in methodology, software and revision. LL was involved in conceptualisation, data curation, funding acquisition, project administration, supervision, validation and revision.

### Peer review

The peer review history for this article is available at https://publons.com/publon/10.1002/1878‐0261.13403.

## Supporting information


**Fig. S1.** Selection of gene signatures for TLS evaluation.
**Fig. S2.** Relationship between tertiary lymphoid structures (TLSs) and clinical information.
**Fig. S3.** Correlation between lymphotoxin α (LTα) and tertiary lymphoid structures (TLSs).
**Fig. S4.** Overexpression of Ltα in SCC7 cells had little influence on the cell condition.
**Fig. S5.** Tongue tumour‐bearing models developed by the injection of SCC7 cells.

**Table S1.** Primers used for qRT‐PCR in this research.

**Table S2.** Comparison between Overall survival and Disease‐free survival among different TLS‐score subgroups in TCGA.

**Table S3.** Comparison between Overall survival and Disease‐free survival among different TLS subgroups.

**Table S4.** Comparison between Overall survival and Disease‐free survival among different TLS and CD8^+^ subgroups.

## Data Availability

The data used to support the findings of this study are available from the corresponding author upon request.
