# Supplementary material for: Tertiary lymphoid structures in head and neck squamous cell carcinoma improve prognosis by recruiting CD8 + T cells
Source: Mol Oncol. 2023 Mar 8;17(8):1514–30. doi: 10.1002/1878-0261.13403 (PMC10399718; doi:10.1002/1878-0261.13403)
Supplement: Supplementary file 1 — Fig. S1. Selection of gene signatures for TLS evaluation. Fig. S2. Relationship between tertiary lymphoid structures (TLSs) and clinical information. Fig. S3. Correlation between lymphotoxin α (LTα) and tertiary lymphoid structures (TLSs). Fig. S4. Overexpression of Ltα in SCC7 cells had little influence on the cell condition. Fig. S5. Tongue tumour‐bearing models developed by the injection of SCC7 cells. [file MOL2-17-1514-s004.zip › mol213403-sup-0006-Supinfo.docx]

**SUPPLEMENTARY TABLES**

**Supplementary Table 1:** The primers used for qRT-PCR in this research.

**Supplementary Table 2:** Comparison of Overall survival and Disease-free survival among different TLS-score subgroups in TCGA.

**Supplementary Table 3:** Comparison of Overall survival and Disease-free survival among different TLS subgroups.

**Supplementary Table 4:** Comparison of Overall survival and Disease-free survival among different TLS and CD8^+^ subgroups.

**SUPPLEMENTARY FIGURES**

**Supplementary Figure 1:** The selection of gene signatures for TLS evaluation. (A) The ROC curves of two different gene signatures for TLS evaluation. The gene signature used for “TLSscore(12)” was derived from (1), including CCL18, CCL19, CCL2, CCL21, CCL3, CCL4, CCL5, CCL8, CXCL10, CXCL11, CXCL13 and CXCL9. The gene signature used for “TLSscore(9)” was derived from (2), including CD79B, EIF1AY, PTGDS, RBP5, CCR6, SKAP1, LAT, CETP and CD1D. The area under the curve of “TLSscore(12)” and “TLSscore(9)” were 0.699 (95%CI, 0.507-0.831) and 0.533 (95%CI, 0.297-0.668). (B) Cox univariate analysis (upper) and Cox multivariate analysis (lower) for TLSscore related overall survival.

**Supplementary Figure 2:** The relationship between tertiary lymphoid structures (TLSs) and clinical information. (A) Cox univariate analysis (upper) and Cox multivariate analysis (lower) for TLS related overall survival. (B) TLS scores in different clinical groups. Patients were divided by lymph node metastasis, clinical stage, pathologic differentiation and invasive pattern. The TLS scores were calculated based on the number of 3 grades of TLS. The error bars represent the SD of the mean. The P values was obtained using the Mann Whitney U-test. (C) CD8^+^ cells in different invasive pattern groups. The error bars represent the SD of the mean. The P values was obtained using the Student’s t-test. (D) The overall survival (OS) and disease-free survival (DFS) curves of patients. Patients were divided into two groups according to CD8^+^ cells count.

**Supplementary Figure 3:** The correlation between lymphotoxin α (LTα) and tertiary lymphoid structures (TLSs). (A) The correlation analysis between TLS score and LTα in TCGA data [R=0.62 (95% CI 0.57-0.67), p<0.001]. (B) The expression of LTα in different TLS related subtypes in TCGA data. The TLS score and subtypes were the same as Fig. 1. The horizontal lines indicate median values, boxes indicate 25% and 75% quartiles, and error bars represent minimum/maximum values. The asterisks indicate the P value: ***< 0.001(Mann Whitney U-test). (C) The mRNA expression of LTα in TLS^-^ (n=9) and TLS^+^ (n=12) patients with HNSCC. The existence of TLS was defined by H&E staining of tissue sections. The error bars represent the SD of the mean. The asterisks indicate the P value: ***< 0.001 (Student’s t-test). (D) Representative pictures of LTα-immunostaining in TLS^+^ and TLS^-^ sections (middle and right, scale bar: 500 μm and 250 μm). The existence of TLS was identified by H&E staining (left, scale bar: 500 μm). The red arrow mark indicates TLS.

**Supplementary Figure 4:** Overexpression of Ltα in SCC7 cells had little influence on the cell condition. (A) Representative pictures of GFP (green fluorescent protein) expression of the transfected SCC7 cells. (B) The mRNA expression of Ltα after transfection. The error bars represent the SD of the mean. The asterisks indicate the P value: ***< 0.001 (Student’s t-test). (C) The result of Cell Counting Kit-8 (CCK-8) assay. (D) Wound healing assay of SCC7. (E) Cell cycle analysis by flow cytometry of SCC7 cells. (F) Apoptosis analysis by flow cytometry of SCC7 cells. The error bars represent the SD of the mean. The P values was obtained using the Student’s t-test.

**Supplementary Figure 5:** Tongue tumour-bearing models developed by the injection of SCC7 cells. (A) H&E staining and immunochemical (IHC) staining of Pan-CK of control group and Ltα group (scale bar: 500 μm). (B) Representative pictures of the IHC staining showed pre-TLS structure on the 12^th^ day taken under 100x (left, scale bar: 500 μm) and 400x (right, scale bar: 125 μm). The pre-TLS was defined as structure where T cells clustered with HEV developed nearby, and B cells started to infiltrate. (C) CD3^+^ cells, CD8^+^ cells, CD19^+^ cells, PNAd^+^ HEV cells control group (n=4) and Ltα group (n=5) on the 12^th^ day. The error bars represent the SD of the mean. The asterisks indicate the P value: *< 0.05; **< 0.01; ***< 0.001 (Student’s t-test).

**REFERENCES**

1. Coppola D, Nebozhyn M, Khalil F, Dai H, Yeatman T, Loboda A, et al. Unique ectopic lymph node-like structures present in human primary colorectal carcinoma are identified by immune gene array profiling. The American journal of pathology. 2011;179(1):37-45.

2. Cabrita R, Lauss M, Sanna A, Donia M, Skaarup Larsen M, Mitra S, et al. Tertiary lymphoid structures improve immunotherapy and survival in melanoma. Nature. 2020;577(7791):561-5.
