# Supplementary figures and images for: Tertiary lymphoid structures in head and neck squamous cell carcinoma improve prognosis by recruiting CD8 + T cells
Source: Mol Oncol. 2023 Mar 8;17(8):1514–30. doi: 10.1002/1878-0261.13403 (PMC10399718; doi:10.1002/1878-0261.13403)

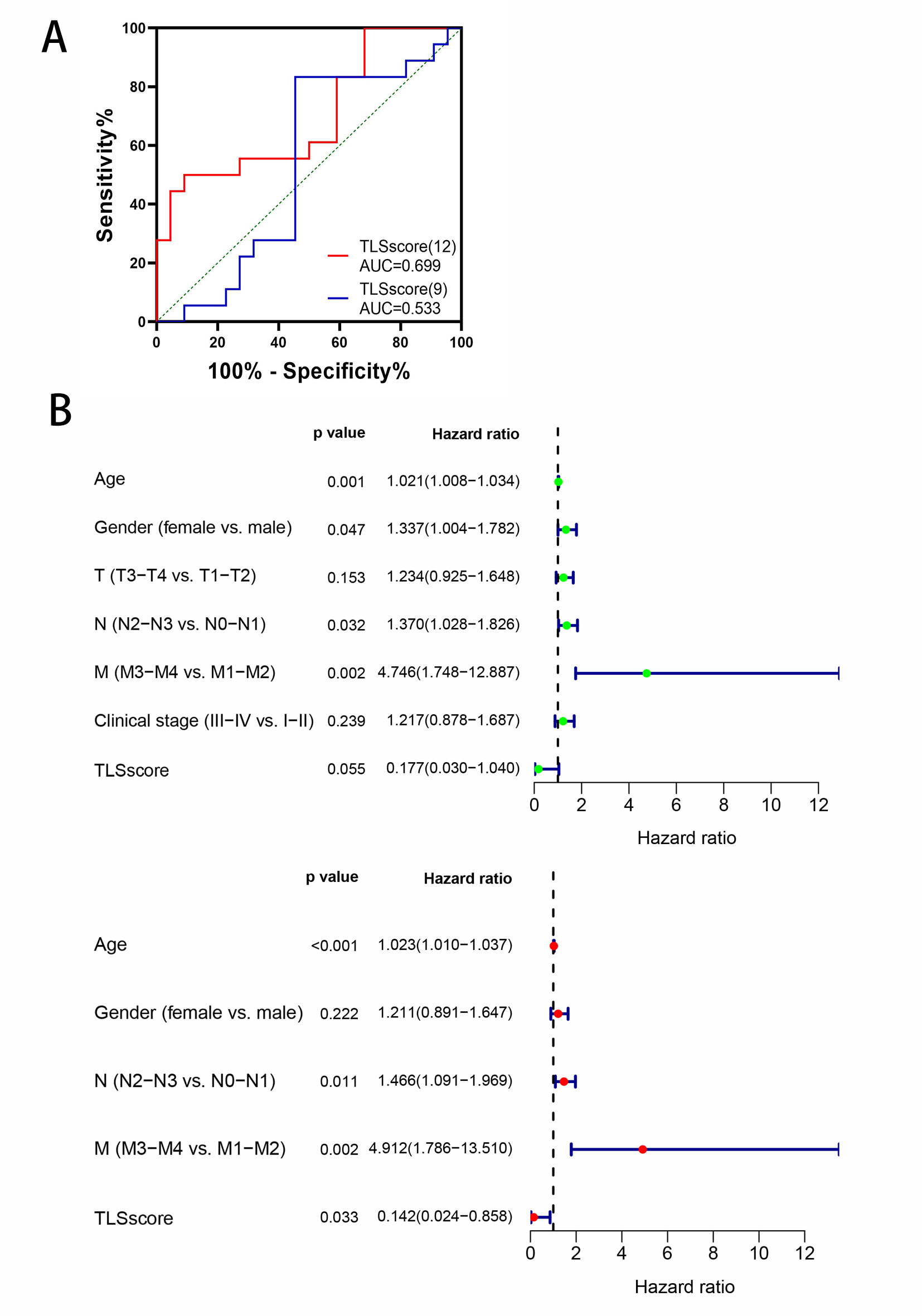

Supplement: Supplementary file 1 — Fig. S1. Selection of gene signatures for TLS evaluation. Fig. S2. Relationship between tertiary lymphoid structures (TLSs) and clinical information. Fig. S3. Correlation between lymphotoxin α (LTα) and tertiary lymphoid structures (TLSs). Fig. S4. Overexpression of Ltα in SCC7 cells had little influence on the cell condition. Fig. S5. Tongue tumour‐bearing models developed by the injection of SCC7 cells. [file MOL2-17-1514-s004.zip › mol213403-sup-0001-FigS1.tif]

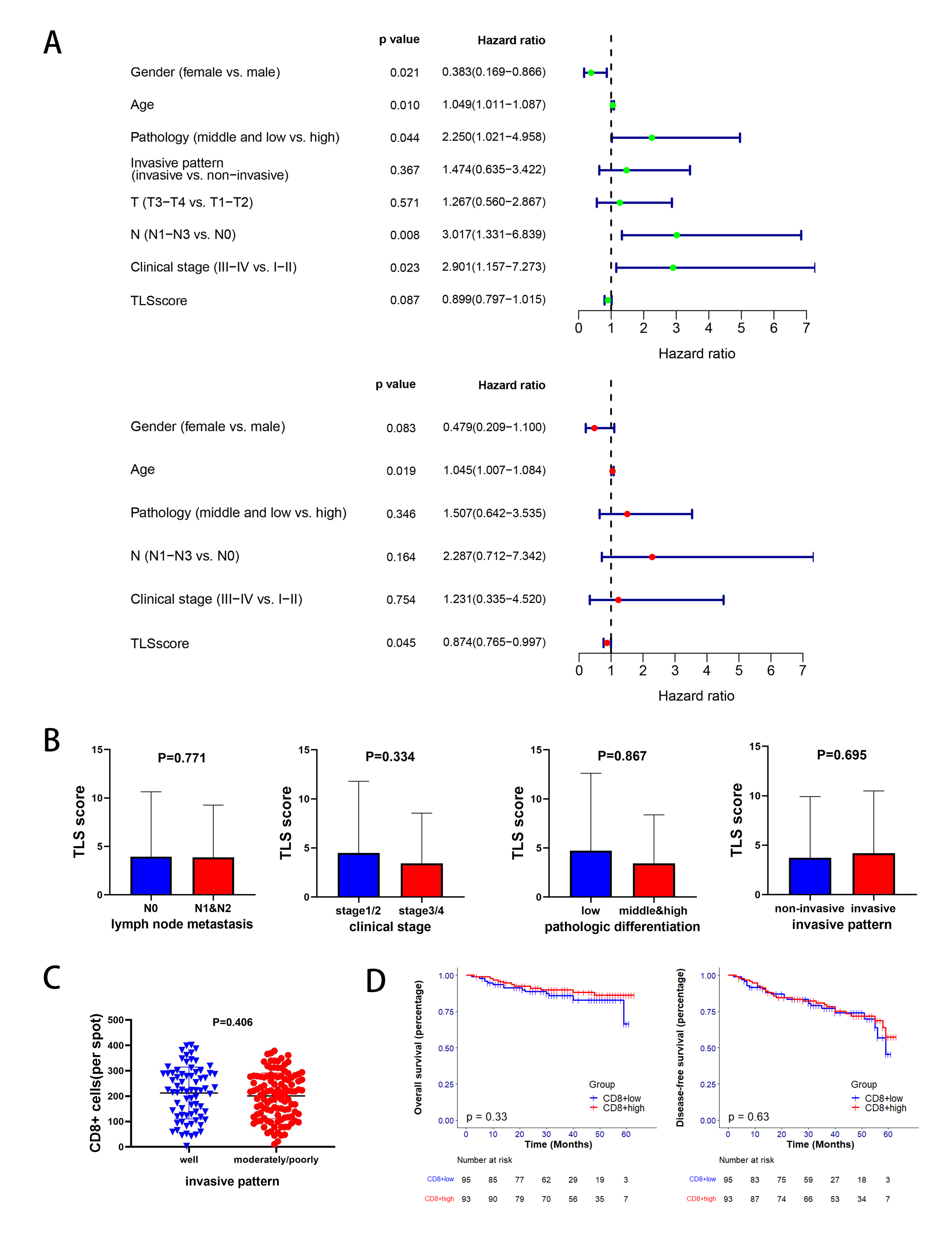

Supplement: Supplementary file 1 — Fig. S1. Selection of gene signatures for TLS evaluation. Fig. S2. Relationship between tertiary lymphoid structures (TLSs) and clinical information. Fig. S3. Correlation between lymphotoxin α (LTα) and tertiary lymphoid structures (TLSs). Fig. S4. Overexpression of Ltα in SCC7 cells had little influence on the cell condition. Fig. S5. Tongue tumour‐bearing models developed by the injection of SCC7 cells. [file MOL2-17-1514-s004.zip › mol213403-sup-0002-FigS2.tif]

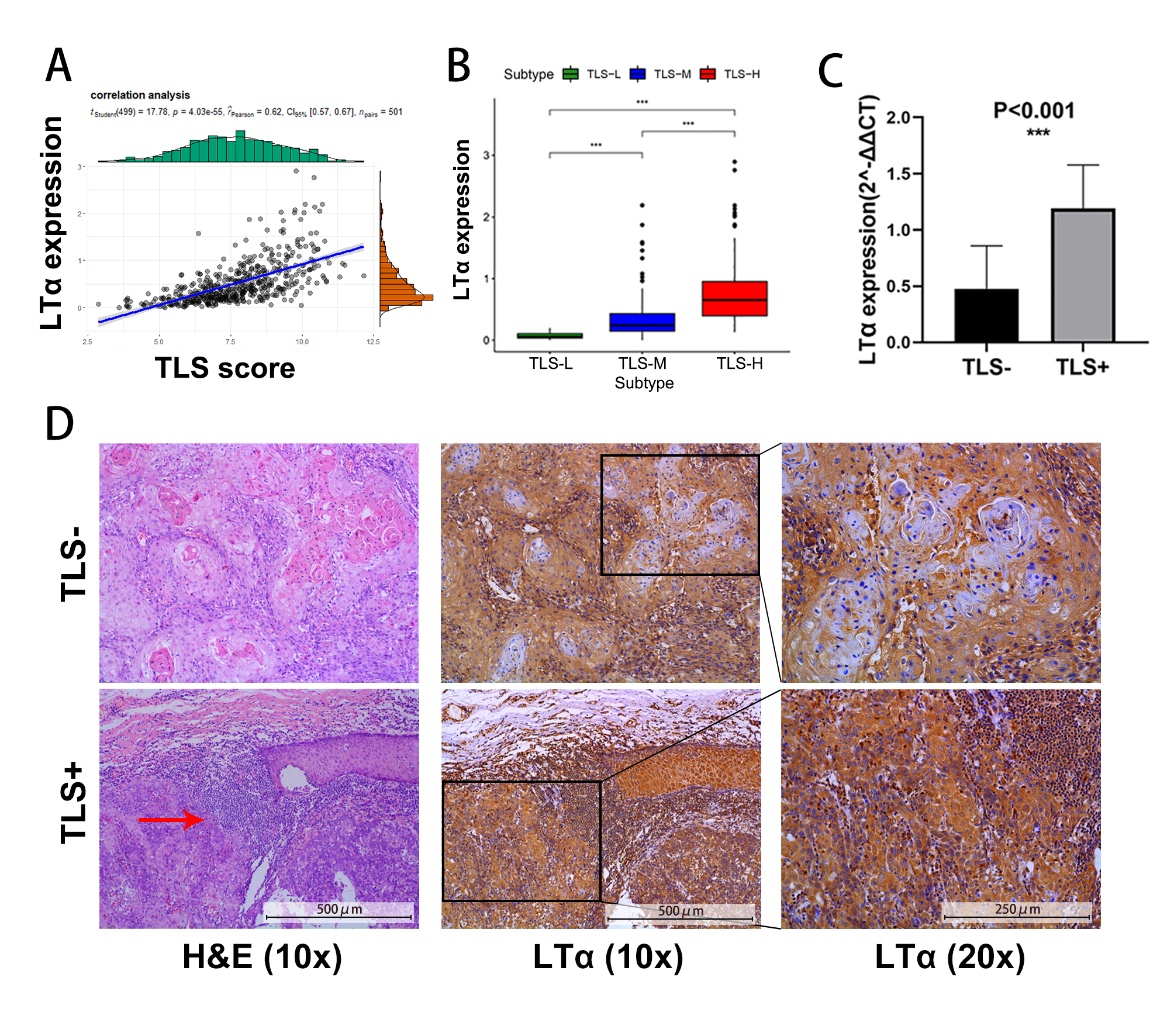

Supplement: Supplementary file 1 — Fig. S1. Selection of gene signatures for TLS evaluation. Fig. S2. Relationship between tertiary lymphoid structures (TLSs) and clinical information. Fig. S3. Correlation between lymphotoxin α (LTα) and tertiary lymphoid structures (TLSs). Fig. S4. Overexpression of Ltα in SCC7 cells had little influence on the cell condition. Fig. S5. Tongue tumour‐bearing models developed by the injection of SCC7 cells. [file MOL2-17-1514-s004.zip › mol213403-sup-0003-FigS3.tif]

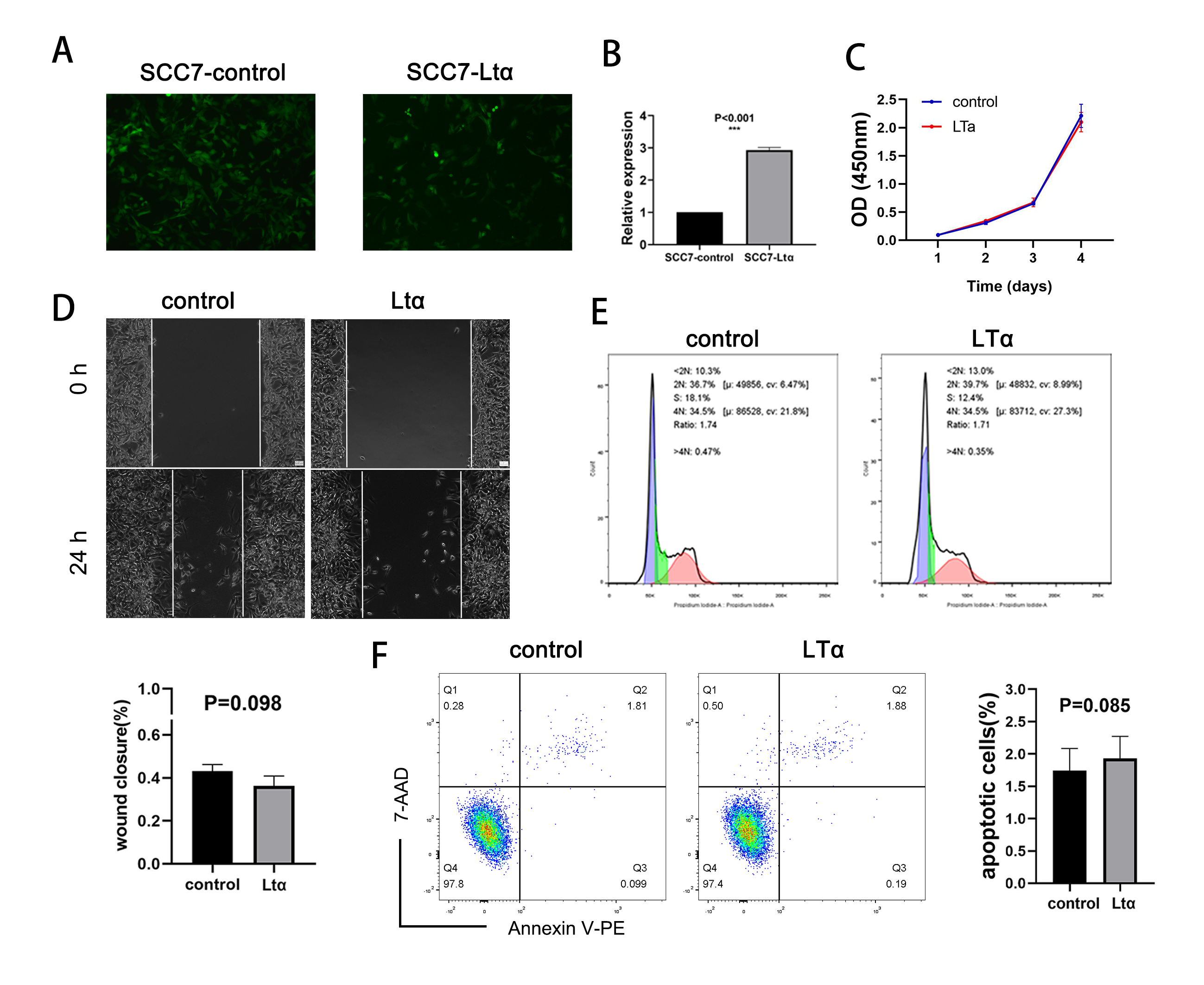

Supplement: Supplementary file 1 — Fig. S1. Selection of gene signatures for TLS evaluation. Fig. S2. Relationship between tertiary lymphoid structures (TLSs) and clinical information. Fig. S3. Correlation between lymphotoxin α (LTα) and tertiary lymphoid structures (TLSs). Fig. S4. Overexpression of Ltα in SCC7 cells had little influence on the cell condition. Fig. S5. Tongue tumour‐bearing models developed by the injection of SCC7 cells. [file MOL2-17-1514-s004.zip › mol213403-sup-0004-FigS4.tif]

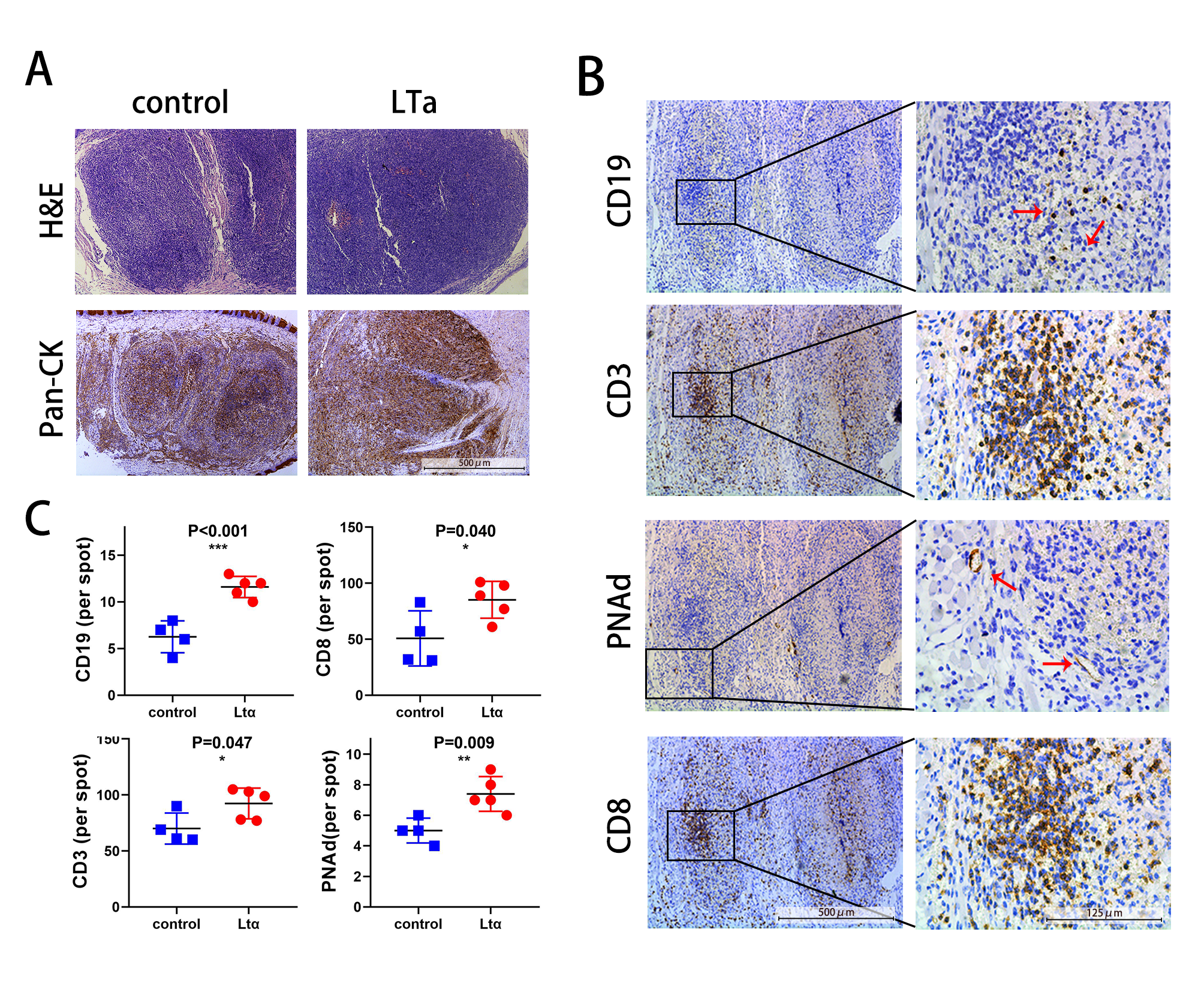

Supplement: Supplementary file 1 — Fig. S1. Selection of gene signatures for TLS evaluation. Fig. S2. Relationship between tertiary lymphoid structures (TLSs) and clinical information. Fig. S3. Correlation between lymphotoxin α (LTα) and tertiary lymphoid structures (TLSs). Fig. S4. Overexpression of Ltα in SCC7 cells had little influence on the cell condition. Fig. S5. Tongue tumour‐bearing models developed by the injection of SCC7 cells. [file MOL2-17-1514-s004.zip › mol213403-sup-0005-FigS5.tif]
