# Supplementary material for: Tertiary lymphoid structures in head and neck squamous cell carcinoma improve prognosis by recruiting CD8 + T cells
Source: Mol Oncol. 2023 Mar 8;17(8):1514–30. doi: 10.1002/1878-0261.13403 (PMC10399718; doi:10.1002/1878-0261.13403)
Supplement: Supplementary file 2 — Table S1. Primers used for qRT‐PCR in this research. [file MOL2-17-1514-s002.docx]

**Supplementary Table 1: Primer sequences for qRT-PCR**

| Primers | Sequences (5’ – 3’) |
| --- | --- |
| GAPDH-F | CAAGGTCATCCATGACAACTTTG |
| GAPDH-R | GTCCACCACCCTGTTGCTGTAG |
| LTA-F | ATGACACCTGAACGTCTC |
| LTA-R | CTACAGAGCGAAGCTCCAA |
| gapdh-f | ACCCAGAAGACTGTGGGGGG |
| gapdh-r | GGATGCAGGGATGATGTTCT |
| lta-f | GACTCTCTGGTGTCCGCTTCT |
| lta-r | GGTACCCAACAAGGTGAGCAG |
| ltb-f | GTACGGGTCGTTATGGTACAC |
| ltb-r | TGACTGATGTTAACGTAGACCC |
| ccl2-f | TTTTTGTCACCAAGCTCAAGAG |
| ccl2-r | TTCTGATCTCATTTGGTTCCGA |
| ccl3-r | AATAGTCAACGATGAATTGGCG |
| ccl3-f | TTGCTGTTCTTCTCTGTACCAT |
| ccl4-f | ACTTCCTGCTGTTTCTCTTACA |
| ccl4-r | CCAAGTCACTCATGTACTCAGT |
| ccl5-f | GTATTTCTACACCAGCAGCAAG |
| ccl5-r | TCTTGAACCCACTTCTTCTCTG |
| ccl8-f | GAATCAACAATATCCAGTGCCC |
| ccl8-r | TTGAGACTTCTGGTCAAGGATC |
| ccl19-f | CCAGGCAACAGAAGGAAGGAACAG |
| ccl19-r | CCAGGGTTTCACGAAGGCAAGG |
| ccl21-f | GTGATGGAGGGGGTCAGGA |
| ccl21-r | GGGATGGGACAGCCTAAACT |
| cxcl9-f | AATCCCTCAAAGACCTCAAACA |
| cxcl9-r | TCCCATTCTTTCATCAGCTTCT |
| cxcl10-f | CAACTGCATCCATATCGATGAC |
| cxcl10-r | GATTCCGGATTCAGACATCTCT |
| cxcl11-f | TCCAAGCAAGCTCGCCTCATAATG |
| cxcl11-r | CAGTCGCCTGTTCTCTGTGTTCC |
| cxcl13-f | TTGTGATCTGGACCAAGATGAA |
| cxcl13-r | GACTTTTGCTTTGGACATGTCT |
