# Supplementary material for: Tertiary lymphoid structures in head and neck squamous cell carcinoma improve prognosis by recruiting CD8 + T cells
Source: Mol Oncol. 2023 Mar 8;17(8):1514–30. doi: 10.1002/1878-0261.13403 (PMC10399718; doi:10.1002/1878-0261.13403)
Supplement: Supplementary file 3 — Table S2. Comparison between Overall survival and Disease‐free survival among different TLS‐score subgroups in TCGA. [file MOL2-17-1514-s003.docx]

Supplementary Table 2. Comparison of Overall survival and Disease-free survival among different TLS-score subgroups in TCGA.

| Groups | Overall survival | | Disease-free survival | |
| --- | --- | --- | --- | --- |
|  | P value | HR (95.0% CI for HR) | P value | HR (95.0% CI for HR) |
| TLS-L versus TLS-M | 0.141 | 1.570 (0.821-3.004) | 0.056 | 1.702 (0.941-3.077) |
| TLS-L versus TLS-H | 0.059 | 1.935 (1.007-3.717) | 0.017* | 2.129 (1.172-3.866) |
| TLS-M versus TLS-H | 0.135 | 1.232 (0.936-1.622) | 0.081 | 1.251 (0.971-1.611) |

The p-values were obtained using the Log-rank test. The asterisks indicate the p-values: *< 0.05.
