# Supplementary material for: Tertiary lymphoid structures in head and neck squamous cell carcinoma improve prognosis by recruiting CD8 + T cells
Source: Mol Oncol. 2023 Mar 8;17(8):1514–30. doi: 10.1002/1878-0261.13403 (PMC10399718; doi:10.1002/1878-0261.13403)
Supplement: Supplementary file 4 — Table S3. Comparison between Overall survival and Disease‐free survival among different TLS subgroups. [file MOL2-17-1514-s005.docx]

Supplementary Table 3. Comparison of Overall survival and Disease-free survival among different TLS subgroups

| Groups | Overall survival | | Disease-free survival | |
| --- | --- | --- | --- | --- |
|  | P value | HR (95.0% CI for HR) | P value | HR (95.0% CI for HR) |
| TLS- versus immature TLS+ | 0.006* | 3.365 (1.329-8.520) | 0.090 | 1.672 (0.924-3.025) |
| TLS- versus mature TLS+ | 0.020* | 8.017 (1.067-60.244) | 0.022* | 3.189 (1.113-9.143) |
| immature TLS+ versus mature TLS+ | 0.396 | 2.383 (0.287-19.796) | 0.227 | 1.908 (0.649-5.611) |

The p-values were obtained using the Log-rank test. The asterisks indicate the p-values: *< 0.05.
