# Supplementary material for: Tertiary lymphoid structures in head and neck squamous cell carcinoma improve prognosis by recruiting CD8 + T cells
Source: Mol Oncol. 2023 Mar 8;17(8):1514–30. doi: 10.1002/1878-0261.13403 (PMC10399718; doi:10.1002/1878-0261.13403)
Supplement: Supplementary file 5 — Table S4. Comparison between Overall survival and Disease‐free survival among different TLS and CD8+ subgroups. [file MOL2-17-1514-s001.docx]

Supplementary Table 4. Comparison of Overall survival and Disease-free survival among different TLS and CD8+ subgroups

| Groups | Overall survival | | Disease-free survival | |
| --- | --- | --- | --- | --- |
|  | P value | HR (95.0% CI for HR) | P value | HR (95.0% CI for HR) |
| TLS+CD8+high versus TLS-CD8+low | 0.007* | 0.199 (0.040-0.993) | 0.106 | 0.644 (0.295-1.405) |
| TLS+CD8+high versus TLS+CD8+low | 0.031* | 0.204 (0.040-1.057) | 0.445 | 0.730 (0.314-1.695) |
| TLS+CD8+high versus TLS-CD8+high | 0.000* | 0.076 (0.016-0.353) | 0.009* | 0.368 (0.170-0.797) |
| TLS-CD8+low versus TLS-CD8+high | 0.163 | 0.382 (0.136-1.078) | 0.266 | 0.571 (0.256-1.273) |
| TLS-CD8+low versus TLS+CD8+low | 0.421 | 1.026 (0.311-3.379) | 0.427 | 1.133 (0.475-2.702) |
| TLS-CD8+high versus TLS+CD8+low | 0.082 | 2.684 (0.896-8.036) | 0.163 | 1.985 (0.834-4.724) |

The p-values were obtained using the Log-rank test. The asterisks indicate the p-values: *< 0.05.
